# Economic and environmental benefits of automated electric vehicle ride-hailing services in New York City

**DOI:** 10.1038/s41598-024-54495-x

**Published:** 2024-02-20

**Authors:** Teng Zeng, Hongcai Zhang, Scott J. Moura, Zuo-Jun M. Shen

**Affiliations:** 1 Department of Civil and Environmental Engineering, University of California, Berkeley, Berkeley, CA 94720 USA; 2 State Key Laboratory of Internet of Things for Smart City, University of Macau, Macao, 999078 China; 3 Faculty of Engineering & Faculty of Business and Economics, University of Hong Kong, Hong Kong, 999077 China

**Keywords:** Civil engineering, Electrical and electronic engineering, Energy grids and networks, Energy and society, Environmental impact, Civil engineering, Electrical and electronic engineering, Energy grids and networks, Energy and society, Environmental impact

## Abstract

A precise, scalable, and computationally efficient mathematical framework is proposed for region-wide autonomous electric vehicle (AEV) fleet management, sizing and infrastructure planning for urban ride-hailing services. A comprehensive techno-economic analysis in New York City is conducted not only to calculate the societal costs but also to quantify the environmental and health benefits resulting from reduced emissions. The results reveal that strategic fleet management can reduce fleet size and unnecessary cruising mileage by up to 40% and 70%, respectively. This alleviates traffic congestion, saves travel time, and further reduces fleet sizes. Besides, neither large-battery-size AEVs nor high-power charging infrastructure is necessary to achieve efficient service. This effectively alleviates financial and operational burdens on fleet operators and power systems. Moreover, the reduced travel time and emissions resulting from efficient fleet autonomy create an economic value that exceeds the total capital investment and operational costs of fleet services.

## Introduction

Transportation electrification and autonomous driving are two major technologies that are reshaping our urban mobility systems. In the past decade, electrification is dominating global transportation trends. The share of electric vehicle (EV) sales globally reached 14% in 2022 and is expected to hit 35% by 2030^1^. Meanwhile, considerable technological and commercial breakthroughs have been achieved in autonomous driving. A prominent initial market for autonomous EVs (AEVs) is ride-hailing services. The worldwide penetration of ride-hailing services is projected to be 17.4% in 2023, generating 312.6 billion USD of revenue^2^. The global autonomous mobility market was valued at $76.1 billion USD in 2020, and is projected to reach $2161.8 billion USD by 2030^3^. Vehicle automation reduces the operation expenditure of fleet companies by cutting labor costs considerably^4^. Furthermore, as advanced eco-driving technologies are designed and deployed, AEVs are expected to achieve high energy efficiency, which further reduces operating costs^5^.

However, the system design and potential effect of AEV ride-hailing services on society are not well understood. Determining the number of AEVs required in this ride-hailing market and supporting infrastructure is critical. The results are consequential for not just researchers but also city planners and policy makers^6^. The optimum number of AEVs and the supporting infrastructure are referred to as fleet management through automation and charging infrastructure planning problems. Many studies have examined a holistic planning and management framework from the perspective of an urban system. Studies can be categorized into two approaches, namely simulation-based and operations research approaches. With the use of simulation tools, studies have focused on congestion effects^7^, congestion pricing^8,9^, fleet re-balancing^10,11^, infrastructure planning^7,11,12^, environmental impact evaluation^13,14^. In the operations research approach, in addition to similar factors^15,16^, vehicle routing^17,18^, fleet sizing^19,20^, pickup and delivery^21,22^ are considered. However, the two approaches exhibit distinct advantages and trade-offs. The simulation-based approach is designed for complex systems, but optimality and deterministic results are difficult to achieve^23^. The operations research approach can be used to compute the social optimum with defined metrics, but it is constrained by the network size. Among these studies, perhaps the most critical breakthrough was proposed in Alonso-Mora et al.^24^ and Vazifeh et al.^25^. In these studies, a graph-theory-based operation approach was adopted, referred to as the minimal path covering problem on a vehicle-shareability network. This method demonstrated a precise approach to intelligently match conventional vehicles with trips and determine the size of a ride-hailing fleet. The approach proved to be computationally effective in a mobility dataset (150M+ trips) from a large network, in the New York Manhattan area. This method is the first that preserves both of the aforementioned advantages and, therefore, can be used as the foundation of this study.

In this study, we propose a tractable mathematical framework to simultaneously address the AEV fleet and charging infrastructure design and management problems in the context of a large urban mobility system. Our research did not specifically target future scenarios (such as changes in travel demand or charging behavior) but rather focused on analyzing a predefined scenario. We expand the network to the entire New York Area, where trips are more widely spread geo-spatially. In the previous study^25^, dense mobility demands within only the Manhattan area shortened the inter-trip connection time. As a result, any vehicle could be matched with a request quickly. Consequently, the effect of fleet size reduction because of vehicle automation within a large area was overestimated. Furthermore, range anxiety from human driver and charging time constraints limit the adoption of EVs. Although range anxiety can be easily addressed with autonomy, the charging downtime of AEVs is considerable. It is critical to account for additional AEVs required to accommodate the loss of service capacity due to charging, which was not well studied in the published literature. In this paper, we first provide insights into the optimal composition of the fleet size, battery size, infrastructure location, and charger capacity. We then conduct a comprehensive techno-economic analysis not only to calculate the societal costs but also to quantify health benefits resulting from reduced emissions. Three insightful findings of this study are as follows:

- Strategic fleet management through automation can enhance vehicle utilization and reduce fleet size by up to 40%. In our simulated experiments, the number of autonomous conventional vehicles reduced from 13,437 to 8100 if proper vehicle dispatch and trip matching are conducted. Furthermore, the unnecessary vehicle mileage traveled (VMT) to scout for the next customers (referred to as the “deadheading” effect) decreased drastically (up to 70%), which alleviated traffic congestion and increased traffic speeds by 4%. This “secondary” effect enables further reduction of the fleet size and facilitates considerable economic benefits. As a result, more than 255 million USD of time cost can be saved annually.
- Unlike the current market trend of large-battery-size EVs, we do not advocate long-range and large power AEVs for ride-hailing systems. With ample charging infrastructure support, a fleet of 9517 cheap 50-kWh AEVs can satisfy the same level of mobility demands as that of a fleet of 8753 expensive 175-kWh AEVs in the New York City region. Detailed economic analysis reflects that using a fleet of 50-kWh AEVs equipped with a network of 50-kW chargers is the socially optimal strategy whose total annual equivalent investment and operation cost is under 95 million USD.
- Electrification and automation jointly provide significant environmental and health benefits. Fleet electrification alone can result in 84% removal of carbon emissions. When both features are considered, over 90% reduction may be achieved. Additionally, optimal fleet management via automation can significantly reduce PM2.5 emissions. The reduced healthcare costs from reduced PM2.5 emissions for the New York City population can be up to 250 million USD/year, which may dominate the aforementioned investment and operation cost of the fleet.

## Results

### Autonomous vehicle utilization

#### Fleet size reduction

**Figure 1:**
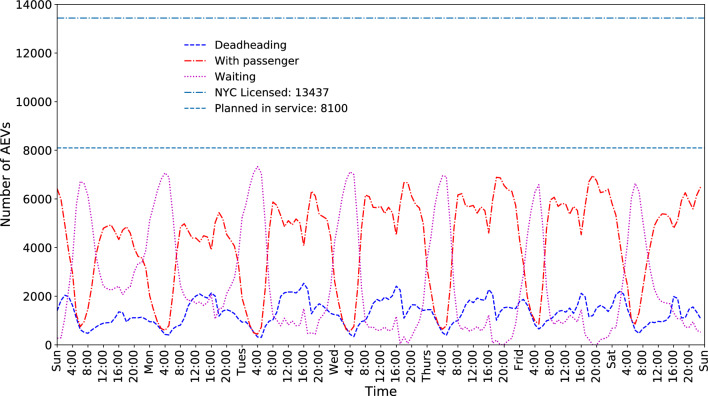
Autonomous conventional vehicle (AV) fleet operation status across a 7-day week. “In service” denotes AVs in service on the road; “Deadhead” is AVs driving to next pickup locations; “With passenger” denotes AVs driving on road with passengers; “Waiting” denotes AVs resting to avoid unnecessary cruising. “NYC Licensed” indicates the total number of licensed taxi (Medallions) in New York City (year 2013), 13,437, whereas with proper fleet automation the fleet size can be reduced to 8100, which is approximately a 40% reduction.

The fleet had 13,437 authorized yellow taxicabs and covers all five boroughs, namely Manhattan, Bronx, Brooklyn, Queens, and Staten Island, including two airports, JFK and LGA^26^. Approximately 90.3% of the pickup events occur within the Manhattan area. The previous study^25^ has revealed that a considerable fleet size reduction (down to 4627 taxicabs) can be achieved with appropriate fleet automation within the region. However, approximately 10% of the mobility demands are outside of this highly concentrated area, which results in long trip connections and increased vehicle deadheading travel. This study^25^ does not recognize that trips within the Manhattan area are close and a vehicle can perform more trips than in other boroughs. Therefore, the results tend to be overpromising for an integrated urban area. We adopted a novel fleet sizing methodology to the entire New York City area to addresses this limitation. The operational status of the fleet across a week is visualized in Fig. 1. From the current 13,437, the fleet size can be reduced to 8100 during peak demands, which is a 40% reduction.

#### Traffic congestion alleviation

Studies have revealed that since the advent of ride-hailing services from 2010, VMT and traffic congestion have increased in metropolitan areas across the world, such as New York City (U.S.)^27^, the San Francisco Bay Area (U.S.)^27^, Shenzhen (China)^28^, Mumbai and New Delhi (India)^29^. This revealed a global concern for increased traffic congestion from ride-hailing services.

**Figure 2:**
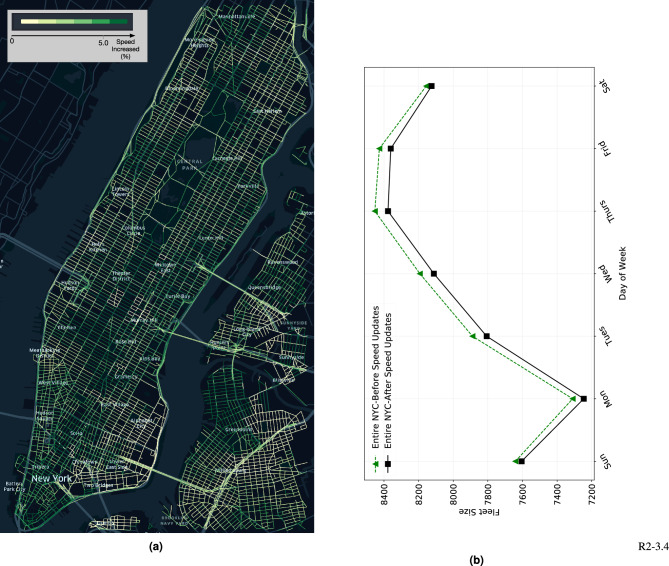
Fleet size reduction on proper fleet automation. The reduced deadheading VMT alleviated traffic congestion. Because of increased average speed, the fleet size could be reduced further. The speed increase across different links in space is detailed in (**a**). The greener the edge is, the higher the percentage of speed improvement it benefits from the AEV fleet. Overall, we observed the biggest benefits to be around the Midtown and Lower Manhattan areas. Furthermore, local streets benefit more from the AEV fleet than highways. (**a**) Spatial speed increase. Figure generated with Kepler.gl (Kepler.gl. Version (0.3.2). Retrieved from https://github.com/keplergl/kepler.gl). (**b**) Fleet size reduction.

This study highlighted the necessity of proper fleet management based on automation, which can potentially reduce traffic congestion. During demand lulls, autonomous vehicles can be rested instead of futile cruising on roads, circumventing deadheading behaviors^27^. They can also be strategically matched to suitable customers by a central operator. This effectively reduced the number of vehicles on the road and the total VMT. Therefore, traffic congestion caused by ride-hailing services can be alleviated through fleet automation. To determine the effect of fleet sizes on the traffic system, we adopted a novel approach to quantify the reduction of the VMT and presented it on the system network. Because VMT dominates the traffic conditions^30^, it was used to quantify traffic volume changes. The fleet autonomy optimization reduced the total traffic volume by 10% and also increased the average system traffic speed from 8.91 to 9.25 m/s, which is a 4% improvement (spatial changes detailed in Fig. 2). Speed increase enabled better vehicle and customer connectivity, that is less vehicles needed for the same demands. Another iteration of fleet size optimization was conducted and we achieved further reduction in fleet sizes (see Fig. 2). On average, 62 vehicles (about 1% of the entire fleet) were eliminated (difference between the green and black curves). This overall finding is particularly relevant to social planners and policy makers.

#### Traffic economic analysis

The alleviation of traffic congestion further enabled a non-trivial economic effect on societal travel time savings. On average, economic savings of more than $255 million a year can be achieved in New York City based on the data in 2013. Furthermore, we conduct a sensitivity analysis on the levels of the household income, detailed in Supplementary Fig. 1. As we expect the annual household incomes to grow from year to year, the economic benefits of an improved traffic system will be more and more profound. As calculated based on the mean household income in the year 2021, the cost saving is estimated to be 528 million USD. Comparing the total value saved to the total annual equivalent financial burden (detailed in the following section), appropriate fleet automation can significant benefit the entire community.

### Effect of battery capacity, charging infrastructure, and charging behaviors

Charging may lead to considerable down times, preventing AEVs from providing mobility services. Therefore, more AEVs are required to satisfy demand. Two factors, namely battery capacity (i.e., driving range) and charging power, simultaneously affect AEV mobility and charging patterns. Specifically, a smaller battery and slower charging speed result in a higher downtime and deteriorates the service capability. The societal cost of AEVs with large batteries and fast charging speeds is substantially higher. Therefore, social planners should meticulously examine and identify the financial “sweet spot”.

#### Effect of battery capacity and charging power on AEV charging behaviors

We conducted geo-spatial and cross-sensitivity analysis with various AEV battery sizes and charging power levels. The battery size ranged from 50 kWh (Tesla Model 3, Nissan Leaf, VW ID.3) to 175 kWh (NIO ET7 and Rivian), and the charging power varied from 25 kW (regular fast charging) to 175 kW (Tesla Supercharger V2). In the simulations, the AEV fleet was set at full energy level as the initial condition. A vehicle was dispatched to charge when there was no sufficient energy to complete the next trip and route to the nearest charging station. When an AEV completed its daily service, it was dispatched to charge back up to the full level, but at a much slower rate (“rest and charge” mode) in preparation for the next day service.

**Figure 3:**
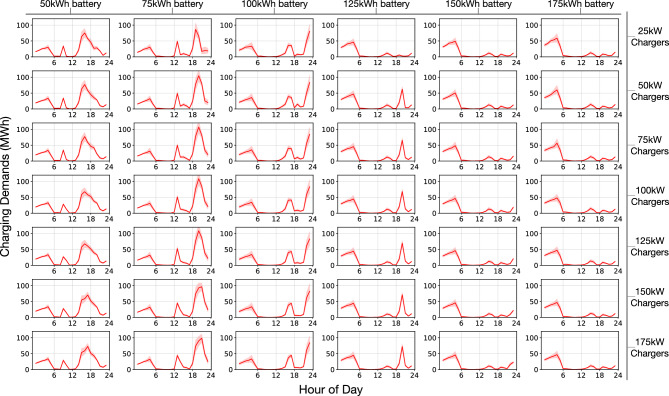
Temporal charging profile variation for combinations of AEV battery size and infrastructure power. Profiles under the same power setting are plotted on one horizontal level; vertical direction varies battery sizes.

The temporal energy demands are plotted in Fig. 3. As displayed, the power level did not affect the vehicles’ charging patterns across the day, but vehicle battery size was influential. As the charging speed varied (same column), obvious changes in charging energy delivered were not observed when peak demands occurred. However, as the battery sizes of the AEVs varied (same row), both the quantity and time at which peaks occurred varied. For small battery sizes, bulk and high charging demands peaked in the afternoon. As the battery size increased, the bulk demands shifted to later in the day. Eventually, with high-energy AEVs, charging sessions only occurred at night.

Range anxiety and downtime due to charging are the two major factors that affect human mobility behaviors. The former influences drivers’ decisions to charge, and the latter induces pure labor cost. It is expensive to leave a driver idle! Whereas for AEVs, who solely comply with control signals, range anxiety and downtime are alleviated. They can properly park at some places and stay idle, or go to a station if the charging criteria is met.

#### Societal economic analysis

**Figure 4:**
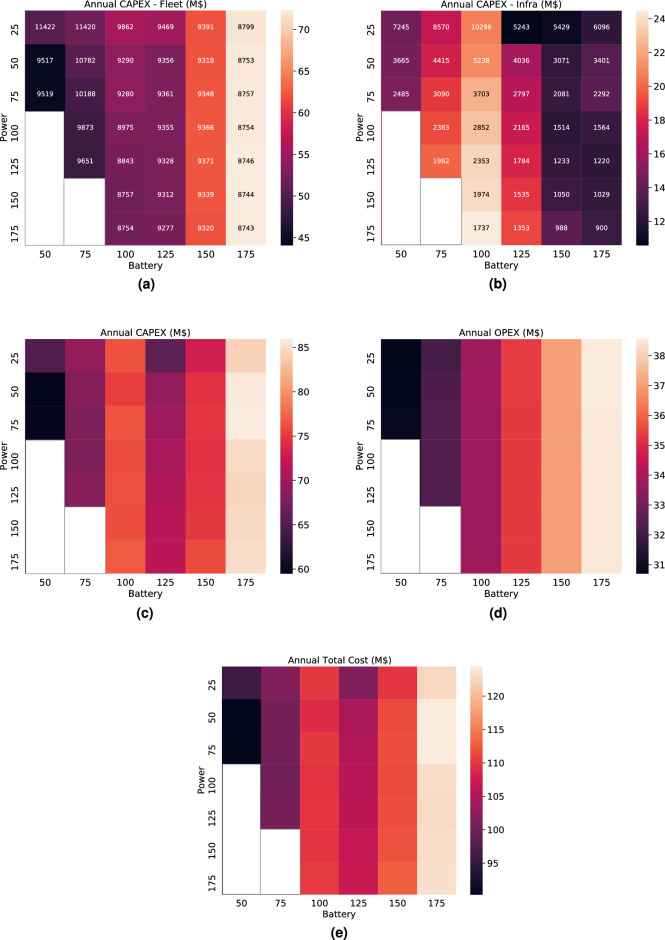
Summary of annual equivalent cost heat map. Top two heat maps (**a**), (**b**) are breakdown of the annual equivalent investment costs (CAPEX, (**c**)). Annotation on each block in (**a**) indicates the number of required AEVs and in (**b**) indicates the number of required chargers. The operation costs in (**d**) include the electricity and maintenance costs posed to the AEV fleet. The summation of (**a**), (**b**) is (**c**) and the summation of (**c**) and (**d**) becomes (**e**). (**a**) Fleet purchase costs. (**b**) Infrastructure placement costs. (**c**) Investment costs. (**d**) Operational costs. (**e**) Total costs.

Economic cross-sensitivity analysis with regard to both the battery size and charging power were performed (Fig. 4). From the perspective of charger powers, some of the resulting charging C-rates (C-rate is a normalized unit of battery charging speed, C-rate = Power/Energy Capacity) may induce considerable damages or degradation effects to the battery packs. The common approach to preserve battery life is to charge at less than 2C-rate^31^. Hence, we intentionally greyed out those (power, battery) combinations with over 2C-rate. The investment cost consists of the capital cost to deploy an AEV fleet of a certain size (Fig. 4a) and to place charging equipment across geo-locations (Fig. 4b). When the AEV is charging, it becomes unavailable for ride-hailing service. As a result, to satisfy certain travel demands, more vehicles need to be added to the fleet. Furthermore, electric vehicles typically spend much longer time refueling than the conventional ones. The longer this down time is, the more trips it misses and requires potentially more AEVs to make up for it. Therefore, both the vehicle battery size and the charging power from the infrastructure play vital roles in dictating the number of extra vehicles to be added. AEV with large battery pack may travel farther but take longer time to recharge whereas AEV with smaller battery may need to charge more than once a day but can go in and out of the station much quickly. A clear gradient is observed in Fig. 4a. When the AEV fleet is equipped with large batteries (175kWh), the recommended fleet size is similar to the number required for AVs. This is intuitive since AEVs with large batteries rarely charge over the course of one day operation. In a scenario with smaller battery AEVs, more charging events happen during the day, resulting in more AEVs needed. This is economical since the increased fleet purchase cost outpaced that of fleet size reduction. As a result, the capital investment cost of the fleet increased accordingly. On the other hand, in Fig. 4b, the trend of the cost of infrastructure placement differed considerably because vehicle battery size dominated the fleet charging pattern. Therefore, for 75- and 100-kWh AEVs, the charging demands were concentrated, which resulted in high peak demands (second and third columns in Fig. 3). Therefore, more chargers are required to accommodate peak demands.

Considering hardware life, the total investment cost is converted to an annual equivalent value through the capital recovery factor and summarized in Fig. 4c. In Fig. 4d, the operational cost is the total annual energy charging cost plus maintenance posed to the AEV fleet. The total annual cost is summed and presented in Fig. 4e. These combinations (50 kW, 50 kWh), (75 kW, 50 kWh) were found to be the most cost-attractive in the planning investment cost analysis (Fig. 4c). In the operational cost analysis after deployment, the smallest battery fleet (50 kWh) was also the most appealing (Fig. 4d). The net effect in Fig. 4e revealed that an AEV fleet of (50 kW, 50 kWh) was the most cost-effective solution, whose annual equivalent cost is under 95 million USD. The total AEV fleet size is recommended to be 9517, which is 15% higher than the AV fleet size.

This result is compelling and non-intuitively. We find that an AEV fleet with smaller battery capacity and a charging network with relatively low charging power minimizes total cost of ownership (TCO). This result flies counter to the trend of developing and deploying chargers with power 250 kW and higher. Meanwhile, high power chargers are concerning for grid operators since their peak power imposes stringent requirements on the local electrical infrastructure.

It’s noted that the current deployment process for charging stations does not consider a budget constraint. The primary focus is on studying the economic feasibility and benefits to meet the travel demands in a given region. However, acknowledging the importance of budget constraints is crucial for practicality in future planning. Considering budget limitations in the deployment process is a sensible approach, as it aligns with the practical considerations faced by city planners and developers. By prioritizing the build plan of charging station locations and sizes based on budget limits, the results will likely be more applicable and useful for those involved in urban development.

### Environmental and health analysis

Implementation of an AEV fleet for ride-hailing services can also provide remarkable environmental and health benefits. On the one hand, converting conventional vehicles to EVs circumvents the use of gasoline fuel and considerably reduces carbon emission. On the other hand, strategic management of an AEV fleet saves unnecessary VMT comparing to unmanaged vehicles with human drivers, which reduces PM2.5 emissions in the urban environment^32^.

#### Effect on environment

**Figure 5:**
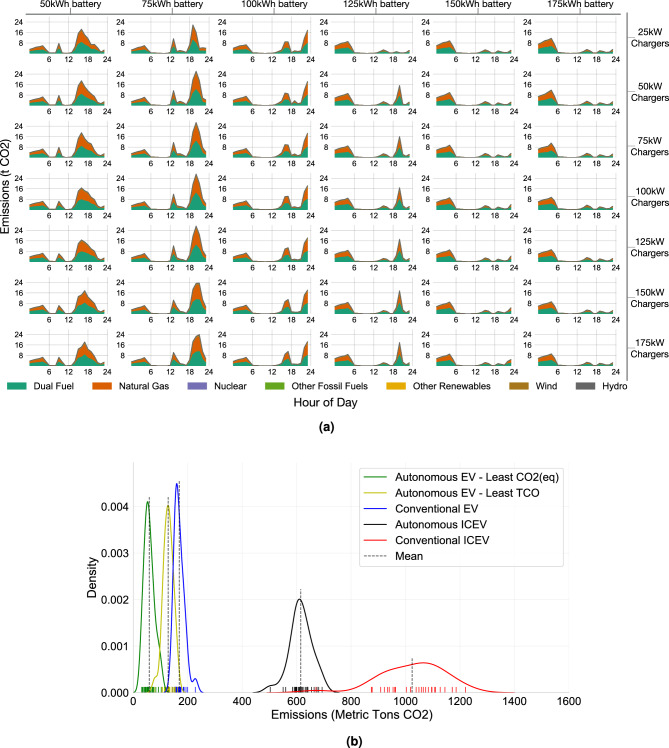

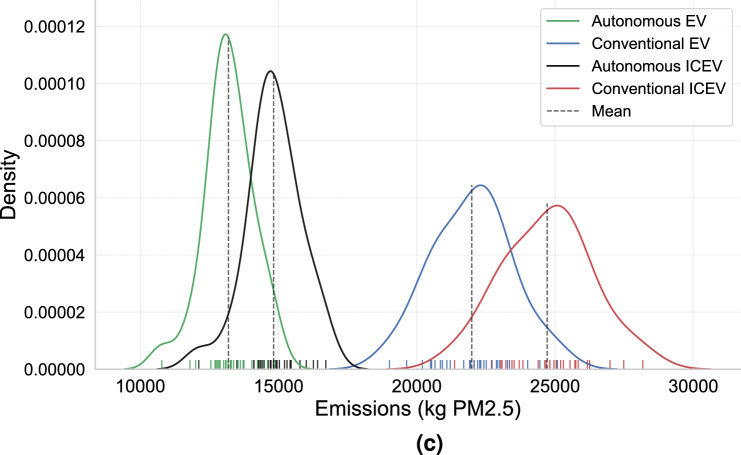
Details of CO_2_ and PM2.5 emissions. Note that CO_2_ emission tightly couples with the fleet’s charging pattern, hence we differentiate two separate scenarios under the “Autonomous AEV”, namely the “least TCO” and the “least CO_2_” (**b**). The “least TCO” scenario does not coincide with the “lease CO_2_” scenario under current grid structure in New York State. Whereas PM2.5 emission tightly couples with the vehicle mileage travelled and thus no case separation is made. (**a**) Temporal CO_2_-equivalent emission profile for various combinations of AEV battery sizes and infrastructure powers. Profiles under the same power setting are plotted on one horizontal level; and the vertical direction denotes the battery sizes. (**b**) Comparative impacts on fleet CO2 emissions due to electrification and autonomy. Comparing the “Autonomous ICEV” and “Conventional ICEV” cases, we observe 40% CO_2_ emission reductions simply by introducing the fleet autonomy strategy. Whereas, comparing the “Conventional EV” and “Conventional ICEV” cases, we observe about 84% changes. Electrification is the primary factor to cut down the greenhouse gas emission. Furthermore, fleet electrification and autonomy together save over 90% CO2 emissions (“Autonomous AEV” vs. “Conventional ICEV”). (**c**) The statistical distributions of the annual PM2.5 emissions particularly due to braking, tires, and road abrasion. For the ICEV fleet, PM2.5 emitted from the tailpipe is also accounted for. We observe that fleet autonomy dominates the reduction effect on PM2.5 emission. Electrification contributes only 11% to PM2.5 reduction. Whereas together with autonomy, 47% of the PM2.5 reduction can be achieved.

We matched the temporal charging profiles with the sources of electricity generation^33^ and calculated the fleet temporal carbon emissions due to energy consumption. These temporal profiles under various combined settings of power and battery are displayed in Fig. 5a. We focused on three prominent scenarios, namely the pre-electrification with conventional vehicles (ICEV), the least TCO, and the least CO_2_(eq) cases, and plotted them in Fig. 5b. For the pre-electrification case, the total CO_2_ emissions were calculated based on the estimated well-to-wheel carbon intensity of gasoline^34^. For the least TCO case, it is identified from the power and battery cross-sensitivity analysis enlisted in Fig. 4e. The optimal combination falls under the setting with 50 kW charging infrastructure and 50 kWh battery AEV fleet. This is a low power and small battery fleet setting. For the least CO_2_(eq) case, the optimal combination of 25 kW charging network and 125 kWh battery fleet is identified from the calculations conducted in Fig. 5a. It does not coincide with the previously identified least TCO case, particularly due to the differences in the AEV fleet charging patterns. The results revealed that natural gas and dual fuel (natural gas plus other fuels, such as oil^33^) generation dominate the emission profile because they are two of the most carbon-intensive generation fuels and constitute more than 39% of the entire generation. Under the current generation mix scenario, fleet electrification alone can result in 84% removal of carbon emissions, where internal combustion emissions are replaced by electricity generated from New York’s electric grid. Automation will further enhance vehicle utilization, reduce VMT, and cut down emissions. When both electrification and automation are considered, over 90% reduction of carbon emissions may be achieved (Fig. 5b).

Notably, in the small-battery fleet, EVs have to be charged multiple times during the day. Fossil fuel generation predominates during the charging down time and thus causes high carbon emissions. However, the penetration of renewable energies is expected to continue and rapidly grow. The New York State announced plans to achieve 70% renewable and a zero-emission electricity by 2030 and 2040, respectively^35^. In the near future, carbon emissions resulting specifically from day charging can be considerably alleviated. Therefore, the most economic beneficial case (middle case in Fig. 5b) may be more environment-friendly than the scenario portrayed in this study.

#### Effect on health

We summarized the annual PM2.5 emissions particularly due to braking, tires, and road abrasion in Table 1 and visualized in Fig. 5c. For the pre-electrified case, PM2.5 emission from the tailpipe was incorporated to the calculation (unit values were summarized in Supplementary Table 1). Note that the U.S. Environmental Protection Agency imposes strict emission regulations on all types of vehicles. It has lowered the amount of pollution light duty vehicles can emit multiple times since the first standards were set in 1970^36^. As a result, the unit PM2.5 emission from tailpipe alone contributes to only 11% of the total emission per kilometer. The majority nowadays comes from braking, tires, and road abrasion. The resulted economic effect on population health was also analyzed. Simply electrifying the fleet of vehicles neither mitigates VMT wasted for cruising nor reduces PM2.5 emissions by significant amount (first and second column of Table 1). The key is to introduce fleet automation and a boost of progress is observed. By deploying a fleet of managed AEVs, over 45% of PM2.5 emission can be saved a year for the New York City. This translates to up to 250 million USD of New York City population health cost reduction per year. More details can be found in Supplementary Fig. 2, where we conducted a Monte-Carlo simulation to measure the economic impacts from the health improvements. We acknowledge that we haven’t accounted for secondary emissions, nor other emissions that cause respiratory health problems, so these calculations are underestimating health impacts. Nonetheless, this analysis provides a compelling reason for social planners and policy makers to transition ride-hailing vehicles to AEV fleets, even with the added infrastructure.

**Table 1:** Total vehicle miles travelled for mobility service and for deadheading (to pick up or relocate) are summarized in the second and third row.

Vehicle type	Conventional ICEV fleet	Conventional EV fleet	Autonomous ICEV fleet	AEV fleet
Total VMT (miles, 5–95% CI)	916,110,061 (818,851,590–1,016,689,156)		549,487,373 (476,396,342–616,159,393)	
VMT with passengers	416,884,181 (360,489,184–465,763,980)			
VMT for deadheading	499,225,880 (458,362,406–550,925,176)		132,603,192 (115,907,158–150,395,413)	
CO_2_ emission (metric tons CO_2_(eq))	1024.18 (875.59–1180.80)	168.85 (146.57–197.62)	615.28 (553.78–678.98)	58.92 (34.31–92.62)
PM2.5 emission (kg)	24,712.99 (22,288.09–27,309.31)	21,986.64 (19,829.27–24,296.54)	14,822.97 (13,341.39–16,357.72)	13,187.70 (11,869.56–14,553.14)

There is a trivial contribution from AEVs being empty and driving to charge. The additional VMT is roughly 0.1% of the VMT for Deadheading. We see more than 70% deadheading mileage reduction with managed fleet. Together we summarize the annual CO_2_ and PM2.5 emission. We have also analyzed the 5- and 95-percentile impact, and given the confidence intervals. To view from left to right, two factors have changed, electrification and fleet automation. The former is in particular beneficial to reduce the CO_2_ emission, whereas the latter contributes most to cut down PM2.5 emission.

## Discussion

The AEV fleet sizing results with automation presented in this work are more practically implied. Comparing to the agent-based simulation approach, these results are more reliable. The optimal fleet sizing and infrastructure placement decisions are deterministic and recommputable. Comparing to the operations research approach, this work first stretches the geographic region. Vehicles tend to serve more trips in the denser area. Our approach not only recognizes the short trips in the denser area (Manhattan), but also account for the longer trips in the more sparse regions (outside Manhattan). This is more geospatially comprehensive. Secondly, this work considers the range constraints and charging downtime, two critical properties to an electric mobility service fleet. The fleet size requirement of a fleet with autonomous conventional vehicles is 15% smaller than the recommended size of an AEV fleet. This demonstrates that the two aforementioned behaviors tightly couple with the AEV fleet’s service capability and hence heavily impact our fleet sizing result. Furthermore, we quantify the secondary traffic impact as a result of our fleet automation strategy. This perspective, which derives from an operations research approach with real urban mobility data, to the authors best knowledge, has not been discussed elsewhere. The AEV fleet can be minimally sized to meet demand, thereby reducing traffic congestion, which produces a feedback loop by increasing traffic speed and enabling further reduction in the AEV fleet size. By optimally designing the AEV fleet size, one can reduce travel times and costs throughout New York City.

The infrastructure planning results also draw important insights. Since AEVs do not experience range anxiety nor time costs, it is not recommended to deploy large battery fleet and super high power charging infrastructure. This counters the current market hypes. In our analysis, we demonstrate that the scenario with large battery fleet and high power chargers may even deteriorate the societal economic benefits. In the most economic viable scenario, the results assume deployment of 3665 DC fast chargers throughout the New York City boroughs. To put into context, New York City has 379 public charging stations, in total 1083 EV supply equipment ports till July 2023^37^. Therefore, to efficiently deploy a fleet of AEVs, it will nearly triple the current size of the charging network. Although there are many incentives to purchase EVs (e.g. state and federal rebates), there are comparatively fewer incentives for deploying charging infrastructure. The recently passed Bipartisan Infrastructure Law will provide $7.5 billion to charging infrastructure development national wide, significantly offsetting the financial burden to stakeholders^38^. Nonetheless, to facilitate widespread deployment of charging infrastructure, we may need incentives that enable shared land use, and increased electrical capacity, in addition to financial assistance. Incorporating the availability and the value of lands and power capacity remains a challenge for conducting relevant studies. These information are important issues that need to be considered by policy makers. To even further enhance efficient charging, an interesting future direction is to incorporate more complex demand forecasting algorithm to achieve global optimality.

Electrification and automation of ride-hailing vehicle fleets provide substantial environmental and health benefits. First, converting New York City’s taxi fleet to AEVs can cut greenhouse gas emissions by over 90%. This is achieved primarily via electrification, where internal combustion emissions are replaced by electricity generated from New York’s electric grid. As the NYISO further decarbonizes their electric generator fleet, the CO_2_ equivalent emissions will continue to reduce. Second, optimally dispatching a New York City AEV ride-hailing fleet reduces PM2.5 emissions by over 45%. The primary reason is not electrification, but rather automating dispatch to substantially reduce VMT. Reduced VMT results in less fine particulate emissions from tires, brakes, and road wear. The reduced PM2.5 emissions has direct impacts on respiratory health, thereby decreasing health care costs by up to 250 million USD per year in New York City. Policies that support electric mobility to reduce air pollution and their health impacts should measure progress by electric VMT, not just the number of EVs sold. Third, to reduce VMT ride-hailing vehicles should park and wait when demand is low. However, increasing parking spaces in New York City for ride-hailing vehicles comes in conflict with other productive uses of land. Further studies should be conducted to evaluate dramatically different city planning designs with transportation infrastructure optimized for ride-hailing AEVs that can park and wait, without consuming otherwise valuable space. It’s also worth pointing out that in the form of future market, ride-pooling (contrary to ride-hailing that has been studied here) can be a further promising solution to reduce deadheading VMTs which then leads to alleviating congestion.

## Methods

### Data

We evaluated various datasets, including the raw mobility data recorded by the New York taxicabs, the transportation network open-sourced by the OpenStreetMap, the electricity generation fuels breakdown by the New York Independent System Operator.

#### Mobility data

The dataset we used contains data of more than 175 million trips a year, or 485,000 a day, for the New York City yellow taxi fleet during 2013. Yellow taxicabs licensed with the *medallions* are the only vehicles authorized to provide mobility services. A total of 13,437 medallions were issued in year 2013.

Four attributes, such as pickup date and time, dropoff date and time, pickup latitude and longitude, and dropoff latitude and longitude, were used to construct trips in this study. A trip $T_i$ is formally defined by an array of four elements, ($t_i^\text {p}$, $t_i^\text {d}$, $l_i^\text {p}$, $l_i^\text {d}$), namely the pickup and dropoff times (capitalized *T* for trip and lower case *t* for time), the pickup and dropoff locations. However, additional post-processing and calculations were required to detail the transportation system conditions, such as the link (from physical intersection to intersection) travel time.

#### Travel time estimation

The mobility dataset provided only the pickup and dropoff locations. The exact path and segment speeds remain unknown. Because the link travel time information is key to the vehicle-shareability network, a large-scale heuristic method was developed^24,25,39^ to compute the estimated link travel time for each traversed road segment {$t_{ud} ~ | ~ \forall (u,d) \in \mathscr {S}$}, where *u* and *d* denote the upstream and downstream intersections of a physical street and $\mathscr {S}$ is the set of all streets. The evaluation criteria was selected such that the average relative error between all estimated and actual trip travel times was minimized. The same method was extended and applied to every hour of the day to determine hourly traffic fluctuations, $\mathscr {T}^\text {travel}$ = {$t_{ud,t} ~ | ~ \forall (u,d) \in \mathscr {S}, \forall t \in \mathscr {T}^{24}$}. An averaged hourly system speed profile is illustrated in Supplementary Fig. 3. When $\mathscr {T}^\text {travel}$ is captured, the travel time between any two intersections *u* and *d* on the map $\mathscr {S}$ at a given time *t* can be determined using a defined routing algorithm, such as the shortest path/time routing. The details of the algorithm and the computation results are provided in Alonso-Mora et al.^24^ and Donovan et al.^39^, respectively.

#### Secondary traffic impact

The proposed framework considerably reduced the number of AEVs required to satisfy given mobility demands. Proper trip assignments and vehicle matching reduced not only the fleet size but also the deadheading mileage. Theses reduced mileage would induce traffic improvement and hence a secondary effect on fleet sizing. Therefore, we proposed Proposition 1. To analyze the secondary traffic effect induced by fleet optimization, we assumed that the change to traffic volume is proportional to the ratio change of the total societal vehicle mileage travelled. However, various types of vehicles contribute differently to the total VMT; this ratio among the private vehicles, taxis, trucks, and others are detailed in Blasio et al.^40^. In 2014, the VMTs of two key sectors, private and taxi vehicles, had a ratio of roughly 5 to 2; and other VMTs were negligible. We leveraged the New York City open source data, the road link volume data, and compared them with the estimated link travel time. Supplementary Fig. 4 shares an example of the volume-time relationship, which is then fitted by a polynomial regression model.

We detailed the process in Supplementary Fig. 5b and specified the link travel time re-estimation as follows:

1. Calculate percentage of VMT change to the total taxi mobility VMT, $\delta _\text {VMT}$;
2. Calculate updated taxi mobility VMT ratio to the system, $\text {VMT}_\text {taxi}^\text {updated} = 2*(1-\delta _\text {VMT})$;
3. Calculate volume change ratio based on Assumption 1, $\delta _\text {vol} = 2/7 - \text {VMT}_\text {taxi}^\text {updated}/(\text {VMT}_\text {taxi}^\text {updated} + 5)$.

Given the output volume impact ratio, $\delta _\text {vol}$, we identified and updated all link travel times based on the fitted regression model.

##### **Proposition 1**

*Reduced unnecessary cruising reduces VMT and alleviates traffic congestion. The resulting traffic conditions further affect the fleet size planning strategies (closed-loop feedback).*

##### **Assumption 1**

The ratio change to traffic volume is proportional to the ratio change to total VMT.

#### Electricity generation profile

AEVs emit zero carbon emission at the end-user level. Thus, the use of AEVs can considerably reduce the carbon footprint of the transportation sector. We traced the energy sources used to generate electricity^33^ and calculated the carbon emission rates associated with various fuels at the hourly basis. The AEV fleet carbon intensity was then calculated according to the hourly fleet-level energy charging demands (Fig. 3).

### Key parameters of the model

The key parameters used to calculate the AEV upfront capital investment costs as well as the fleet automation operation costs are summarized in Supplementary Table 1. Environmental and health-related parameters, which are used for quantifying emission and health effects, are also included.

### Fleet sizing algorithm

The contributions from Vazifeh et al.^25^ were considered in this study. The shareability network it proposed discussed the sharing of vehicles to connect and complete multiple trips. Our network methodology expanded this model but was more comprehensive and customized toward the AEV operations. The model considers the EV range constraints and downtime effect of the autonomous machines, both of which are essential for AEV fleet analysis.

#### Vehicle-shareability network

The vehicle-shareability network is based on a directed acyclic graph $V = (\mathscr {N},\mathscr {E})$ to describe the relationships among trips $\mathscr {T}$. As illustrated in Supplementary Fig. 6, a vertex $n_i \in \mathscr {N}$ in the graph represents a ride-hail/mobility request $T_i \in \mathscr {T}$, and an edge represents a feasible connection between two trips performed by the same vehicle in time sequence. Formally, considering two consecutive trips $T_a$ and $T_j$ with given dropoff and pickup time ($t_a^\text {d}$, $t_j^\text {p}$) and the travel time between ($l_a^\text {d}$, $l_j^\text {p}$), $t_{aj}^\text {conn}$, an edge can be connected on the graph only if $t_j^\text {p} - t_a^\text {d} \ge t_{aj}^\text {conn}$. Therefore, the edges indicate feasible paths for subsequent trips. A chain of connected nodes indicates the trip chain to be traveled by a vehicle. The minimum fleet size problem can then be translated into a minimum path cover problem. Studies have revealed that this problem is equivalent to the maximum matching problem on bipartite graphs, which can be solved by the Hopcroft–Kap algorithm in polynomial time $O(|\mathscr {E}||\mathscr {N}|^{1/2})$^25^. This approach enabled computational efficient algorithms to realize precise and optimal solutions.

#### Fleet size without range constraints

First, we started with AEVs of sufficiently large battery capacity. An ideal scenario without range constraints in mobility service was considered. This assumption is an equivalent scenario to operate the autonomous vehicles with convention internal combustion engines.

##### Stage 1. Limiting trip connection time

To reduce model complexity, the intertrip travel time $t_\delta$ was introduced. This more stringent connection time reduced the number of graph edges in Supplementary Fig. 6. It has practical implications. When $t_\delta$ becomes infinitely large, the sequenced trips that are distant (in space and/or in time) can be connected. However, it wouldn’t be ideal to construct such a trip chain. It leads to long and unnecessary travel-to-pickup distances, results in worse emissions and exacerbates the traffic congestion. A candidate solution like this becomes trivial. Hence, in our setting, two trips are connected only if two conditions are satisfied, namely $t_\delta \ge t_j^\text {p} - t_a^\text {d} \ge t_{aj}^\text {conn}$ (Supplementary Fig. 7). In our experiment, $t_\delta$ was set to 15 min (It was shown from the previous study^25^ such that for allowable connection time $t_\delta$ more than 15 min, the effects on the fleet size and mobility service were negligible). The assumption introduced is therefore no passenger waiting is considered; yet it can be easily revised and extended for other applications.

##### Stage 2. Relaxing waiting time

To enable a further stage complexity control, we detailed the main behavioral difference between an AEV and a human-driven taxi as follows: allowing an AEV wait has lower economic burden than that of a human driver, who is paid hourly wage ($21.07/h for Uber driver^41^ and $14.77/h for New York taxi driver^42^). The typical daily mobility demand patterns correspond to two peaks, one early in the morning (before work) and the other late in the afternoon (after work). Therefore, we first combined solved trip chains from Stage 1 as the pseudo-trips and introduced another parameter of time limit $$t^\text {down}$$ (we set to 600 min, any long enough horizon should suffice) to reconstruct the shareability network (Supplementary Fig. 8). An edge can be connected only if $$t_\delta + {t}^\text {down} \ge t_j^\text {p} - t_a^\text {d} \ge t_{aj}^\text {conn}$$. Thus, some AEVs can be parked and deactivated into the “sleep” mode during the period of demand trough and then be reactivated to when the second demand peaks (Supplementary Fig. 9). Therefore, a further minimization of the fleet size can be achieved.

#### Fleet size with range constraints

We introduced the range constraint to the AEV fleet by introducing the AEV battery capacity limit *B*.

*Step 1. Warm start without range constraints*

This is the same step as mentioned previously. The outputs were the chains of trips $\mathscr {T}^\text {chains}$, each to be served by a conventional autonomous vehicle.

*Step 2. Identify charging events in trip chains*

We consider the given battery capacity *B*, energy efficiency $\eta$, and convert it to the effective driving range $L^\text {DR}$ using the following formula:

1 $$\begin{aligned} L^\text {DR} = \frac{B}{\eta }. \end{aligned}$$

By considering the identified trip chains $T_i^\text {chain}$ in $\mathscr {T}^\text {chains}$, we identified the exact times and locations the charging events occur. The AEVs charge in three scenarios. First, when an AEV does not contain sufficient energy to perform the next trip or travel to the next charging station. The second is the “smart heuristic” scenario when an AEV has a considerably large time window until the next upcoming trip. Mathematically, $t_j^\text {p} - t_a^\text {d} \ge t^\text {down-charging}$ ($t^\text {down-charging}$ is set at 30 min) is satisfied between two trips $T_j$ and $T_a$. Notably, $t^\text {down-charging} > t_{aj}^\text {conn}$, namely the period until the AEV needs to travel to the next pickup location is sufficiently long and is flexible to charge. The final situation is the “recovery” scenario in which by the end of the vehicle trip chain, the AEV is dispatched to recover its consumed energy, for the next-day mobility service.

*Step 3. Reconstruct vehicle-shareability network*

After Step 2 identification for each AEV *i*, we combined all upstream trips before the first identified charging event at $t_i^\text {charge}$ as one super pseudo trip, $T_i^\text {S-pseudo}$ = {$T_i ~ | ~ t_i^\text {d} \le t_i^\text {charge}$, $\forall T_i \in \mathscr {T}_i^\text {chain}$} (Supplementary Fig. 10). We could re-chain all beforehand trips because any newly identified charging event at any time $t^\text {charge}$ does not affect the upstream trips that already occurred. Thus, a novel vehicle-shareability network was constructed and all the subsequent trips were reset and re-optimized.

*Step 4. Re-solve the fleet sizing problem*

We re-solved the problem to determine a new set of trip chains, $\mathscr {T}^\text {chains}$. Next, a ${T}_i^\text {chains} \in \mathscr {T}^\text {chains}$ may look like: {$T_i^\text {S-pseudo}$, $T_{i,x}$, $T_{i,y}$, $T_{i,z}$, $\dots$}, where $t_i^{\text {S-pseudo},\text {d}}$, $t_{i,x}^\text {p}$, etc. respect the relevant conditions (Supplementary Fig. 11).

We summarized the overall algorithmic flowchart in Supplementary Fig. 5a. This iterative algorithm can still be solved using the polynomial expression, with complexity $O(|T||\mathscr {E}| |\mathscr {N}|^{1/2})$. Recall that the Hopcroft–Karp algorithm preserves a complexity of $O(|\mathscr {E}| |\mathscr {N}|^{1/2})$ for solving the maximum matching problems in bipartite graphs. The key of our algorithm is that only *O*(|*T*|) iterations are required to combine and re-solve trips. Therefore, this algorithm guarantees termination. Furthermore, the algorithm typically converges considerably faster than the total number of time steps (Supplementary Fig. 12). The intuition behind is AEV fleet charging need does not incur in every iteration. Only when a charging need is detected, would the algorithm reconstruct the vehicle shareability network and introduce new AEVs to dispatch. Secondly, the fleet size generally stabilizes after the peak hours. Hence, the algorithm converges much faster than the total number of time steps. The program may be pre-maturely terminated to accelerate the process as the number of the newly identified charging events yield an upper bound to the additionally required AEVs and the total fleet size.

### Charging infrastructure planning

The output results of the aforementioned algorithm include all spatial and temporal information of the charging events. We adopted the classic K-means clustering method to determine the locations where charging infrastructure could be placed. This methods proved to be meaningful and computationally efficient^43^. In this paper, the average travel distance to the charging sites was capped at 1 mile, to ensure the chargers were conveniently accessible to most AEVs. In addition, a probabilistic constraint was adopted to ensure that 95% of the charging events may reach a station within 2 miles.

We applied another probabilistic constraint to determine the number of required chargers at each charging station. This constrained programming embedded a service-level model^43^, which is subject to a tunable quality of service parameter $\alpha$ (we set $\alpha = 80\%$ in our experiments). Thus, the model guaranteed that under ($\alpha \times 100$)% of the time, an AEV was charged immediately upon arrival. Given the average charging requests $\lambda _{tk}$ at location *k* during hour *t*, the minimum number of chargers $n_k$, was constrained as follows:

2 $$\begin{aligned} n_k&\ge t^\text {charge}\lambda _k + \Phi ^{-1}(\alpha ) \sqrt{t^\text {charge}\lambda _k}, \end{aligned}$$

3 $$\begin{aligned} \lambda _k&= \max _t \lambda _{tk}, \end{aligned}$$

where $t^\text {charge}$ is the charging duration to full and $\Phi ^{-1}$($\cdot$) is the inverse of the cumulative distribution function of the standard normal distribution. As Eq. (2) indicates, the number of chargers is dictated by the expected peak hour charging demands and the arrival rate $\lambda _k$ (first term); it is additionally affected by the excessive demands over the mean (second term). This phenomenon is covered in detail in previous studies^43^.

A visualization of an example for the geo-spatial distribution of chargers is presented in Supplementary Fig. 13. In this particular scenario (50 kWh battery AEVs and 50 kW chargers), a total of 3,665 chargers were required to satisfy all AEV charging demands.

### Calculation of total cost

The total annual equivalent cost (Fig. 4) is calculated as follows.

4 $$\begin{aligned} C^\text {total} = \underbrace{\underbrace{CRF^\text {veh} * C^\text {veh} + CRF^\text {batt} * C^\text {batt}}_{\text {Fleet Cost in Fig.} 4a} + \underbrace{CRF^\text {infra} * C^\text {infra}}_{\text {Infrastructure Cost in Fig.} 4b}}_{\text {CAPEX in Fig.} 4c} + \underbrace{C^\text {oper}}_{\text {OPEX in Fig.} 4d}, \end{aligned}$$

where all the capital recovery factors (*CRF*s) are calculated by the following equation:

5 $$\begin{aligned} CRF&= \frac{i(1+i)^n}{(1+i)^n-1}, \end{aligned}$$

in which *n* is the number of years and *i* is the discount factor. For $CRF^\text {veh}$ and $CRF^\text {infra}$, the time horizon $n=20$ years (Supplementary Table 1); whereas for $CRF^\text {batt}$, the time horizon depends on AEVs’ charging behaviours.

Batteries inevitably undergo degradation because of charging. Small battery fleet tends to charge more frequent in a day and hence may degrade quicker. In our calculation (Fig. 4), we consider the cycle life of the lithium-iron phosphate batteries, which are widely adopted in the current EV models. We also conservatively estimate the battery degradation cost by assuming at worst case 1700 cycles. This corresponds to the lithium-iron phosphate batteries performing at 0 °C temperature^44^. For each operation scenario (power, battery combination), we group the AEVs based on their daily charging frequencies. As a result, for each group of batteries with similar charging frequencies, there is a different *n*, calculated as follows.

6 $$\begin{aligned} n^\text {batt}&= \frac{\text {cycle life}}{\text {daily charging frequency} * \text {365 days/year}}. \end{aligned}$$

### Calculation of travel time cost

A common method in literature to measure the cost of travel time is presented in Gwilliam^45^ and Litman^46^. The hourly cost of travel time is equal to 30% of a household income per hour. The median value of New York household annual income in 2013 (the same year with the ride-hailing data analyzed in this paper) is reported to be $53,843^47^. We then converted the value to an hourly basis and multiplied it with the estimated total amount of time saved. The formulas to estimate the total economic effect is as follows:

7 $$\begin{aligned} \text {Total time saved}&= \text {Total VMT} / \text {speed before} - \text {Total VMT} / \text {speed after} \approx 35 \text {Million hours}, \end{aligned}$$

8 $$\begin{aligned} \text {Societal savings}&= \text {Total time saved} \times 30\% \text { household hourly income} \approx \$255 \text {Million}. \end{aligned}$$

The annual total vehicle mileage travelled data is from the New York State Department of Transportation Office of Technical Services. The value increases from 18,759 million miles in 2016 to 18,944 miles in 2019, less than 0.1% increment. We assume annual travel pattern of this society remain the same level. Therefore, on average economic savings of more than $255 million a year can be achieved in New York City based on the data in 2013.

### Calculation of population economic impact of PM2.5 emissions

We adopted the “effect factor” (EF)^48^ as a measure of the relationship between the population intake of pollutants, like PM2.5, and the associated health effects. It describes the correlation between the change in mortality to the change in mass of PM 2.5 inhaled. The change in mortality is then converted to an economic measure by multiplying with the value of statistical life-year (VSLY). VSLY, converted from the value of statistical life, is the annual monetary amount that people are willing to pay for small reductions in their mortality risks^49^. The numeric values used in the study are recorded on Supplementary Table 1.

The population economic impact of emissions due to PM2.5 is calculated as follows:

9 $$\begin{aligned} \text {Total PM2.5 emission}&= \text {Total VMT} \times \text {PM2.5 emission/mile}, \nonumber \\ \text {Population intake (PI)}&= \text {Intake fraction (IF)} \times \text {Total PM2.5 emission}, \nonumber \\ \text {Population economic impact}&= \text {PI} \times \text {EF} \times \text {VSLY}. \end{aligned}$$

### Supplementary Information

Supplementary Information.

## Data Availability

Yellow cab trip record data are available from the dataset publicized by the New York City Taxi & Limousine Commission: https://www1.nyc.gov/site/tlc/about/tlc-trip-record-data.page.
