# Supplementary material for: Economic and environmental benefits of automated electric vehicle ride-hailing services in New York City
Source: Sci Rep. 2024 Feb 20;14:4180. doi: 10.1038/s41598-024-54495-x (PMC10879127; doi:10.1038/s41598-024-54495-x)
Supplement: Supplementary file 1 — Supplementary Information. [file 41598_2024_54495_MOESM1_ESM.pdf]

Supplementary Information for Economic and Environmental Benefits of Automated Electric Vehicle Ride-Hailing Services in New York City

Teng Zeng[1], **Hongcai Zhang**[2*], **Scott J. Moura**[1], **and Zuo-Jun M. Shen**[1,3]

[1]Department of Civil and Environmental Engineering, University of California, Berkeley, Berkeley, CA 94720, USA
[2]State Key Laboratory of Internet of Things for Smart City, University of Macau, Macao, 999078, China
[3]Department of Industrial Engineering & Operations Research, University of California, Berkeley, Berkeley, CA 94720, USA
[*]hczhang@um.edu.mo

ABSTRACT

This file includes: Supplementary Figures 1-13, Supplementary Table 1.

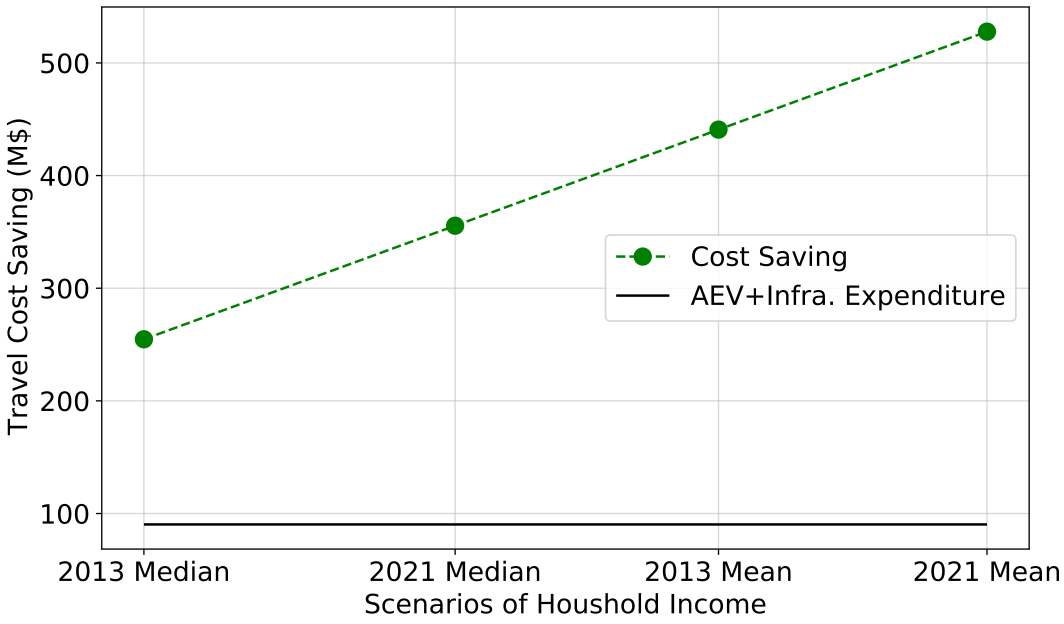

Supplementary Figure 1. Cost savings estimated from reduced travel time (Million USD) in four scenarios. Namely, these scenarios are based on different levels of household income. The value we extracted from[1–3] are $53,843 (2013 median household income), $75,157 (2021 median household income), $93,196 (2013 mean household income), and $111,583 (2021 mean household income). The calculated values from these scenarios are reported to be at least 254 million USD. As we expect the annual household incomes to grow from year to year, the economic benefits of an improved traffic system will be more and more profound. As calculated in the "2021 Mean" scenario, the cost saving is estimated to be 528 million USD, far dominating the expected annual costs to deploy an AEV fleet for New York City (black solid line).

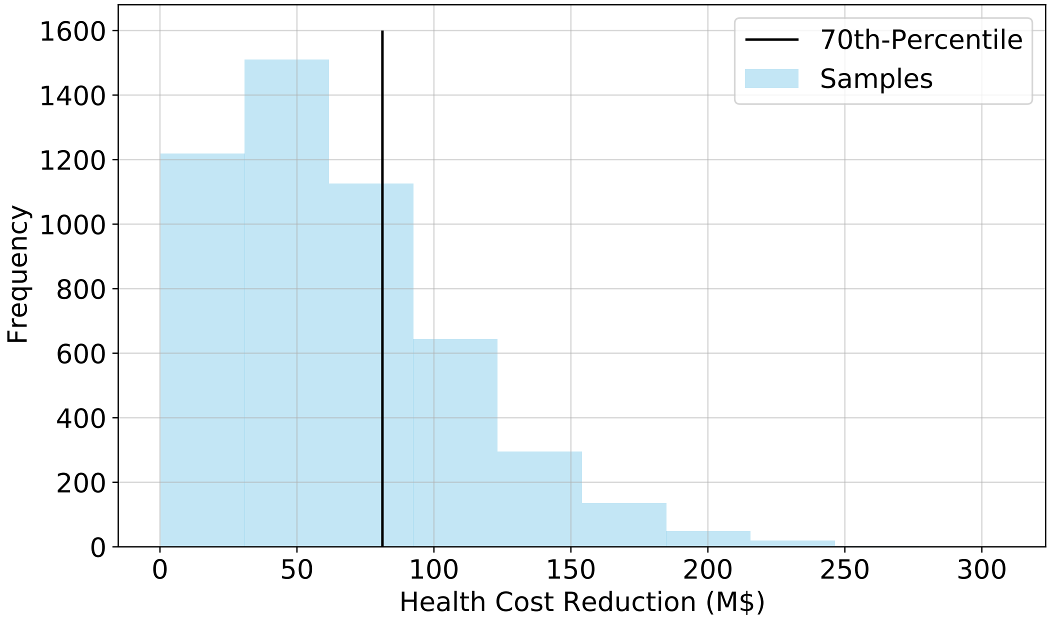

Supplementary Figure 2. Population health cost reduction (Million USD) based on the converted Value of Statistical Life Year (VSLY) sampled from a Weibull Distribution, estimated from 26 related studies recommended by the U.S. Environmental Protection Agency[4]. The Value of Statistical Life (VSL)[4] are converted to Value of Statistical Life Year (VSLY) considering inflation by $VSLY = \frac{rVSL}{1-(1+r)^{-L_{\bar{a}}}}$[5], where $-L_{\bar{a}}$ is the average number of remaining life years for the average person in the sample and r represents the discount rate. At 70-percentile of the samples, the health cost is around 81 Million USD, which compensates the cost to deploy the AEV fleet completely. Under certain scenarios as we expect the value of statistical life to grow, the value to health improvement dominates the basic costs.

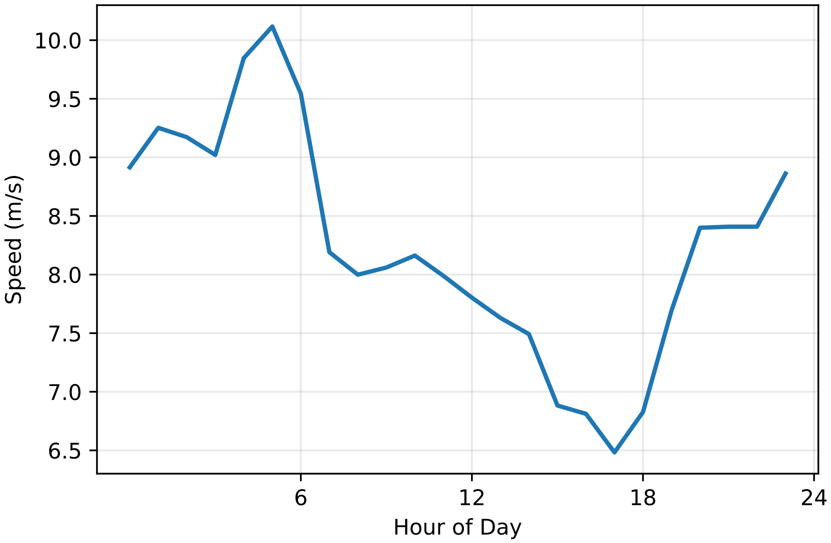

Supplementary Figure 3. Averaged hourly system speed.

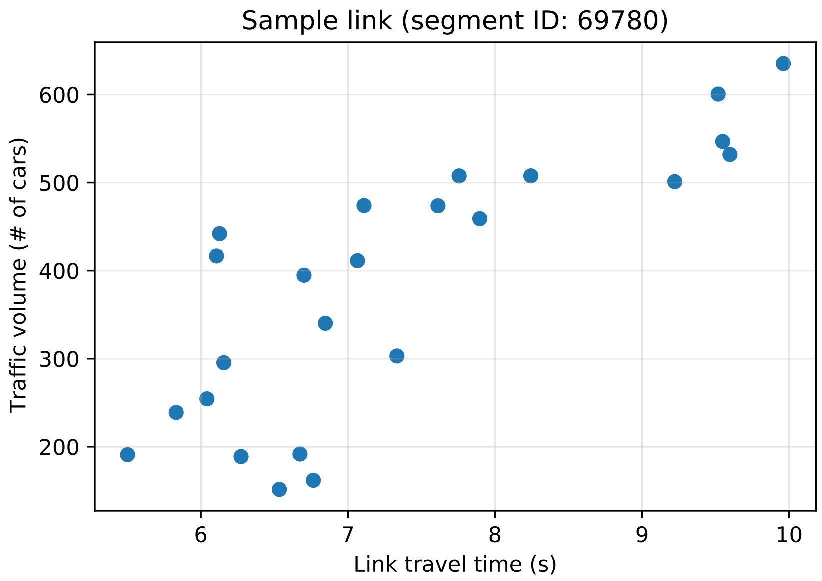

Supplementary Figure 4. Cross-matched link volume-travel time graph.

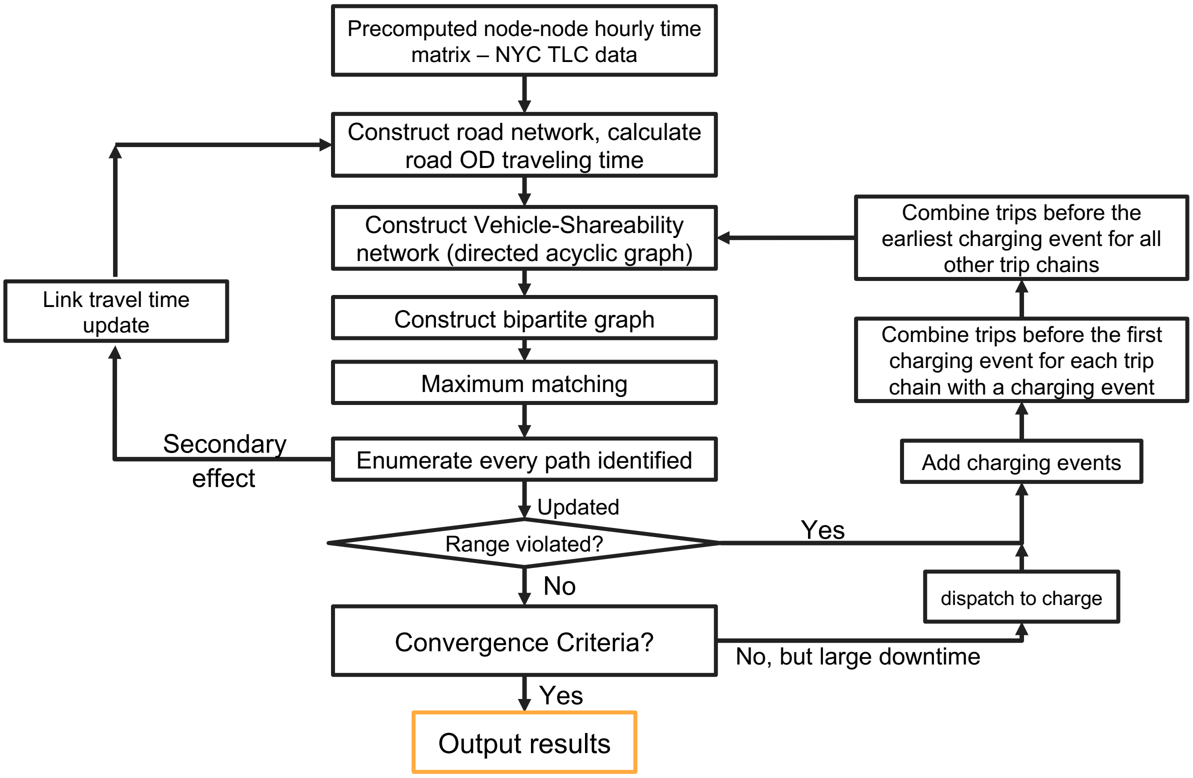

(a) Overall algorithmic flowchart, solvable in polynomial time $O(|T||\mathscr{E}||\mathscr{N}|^{1/2})$.

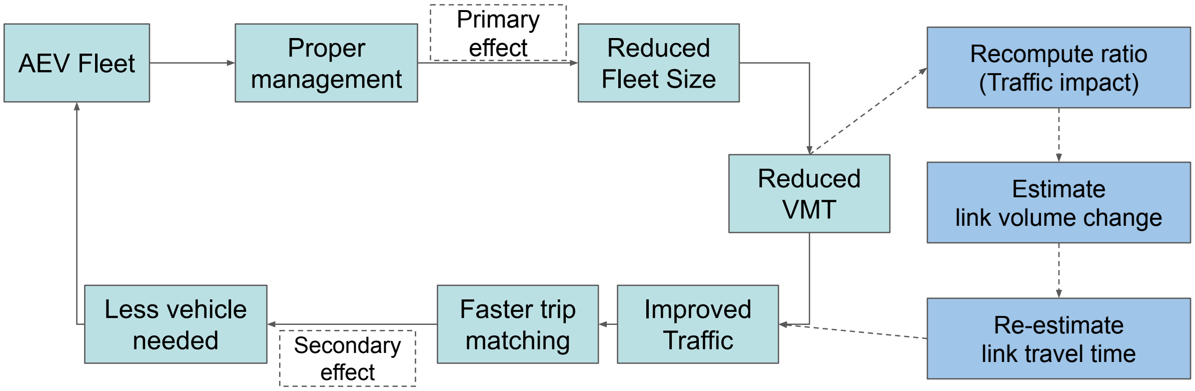

(b) Secondary traffic effect and link travel time update flowchart (expands from the left loop in Fig.5a above).

Supplementary Figure 5. Algorithmic flowchart.

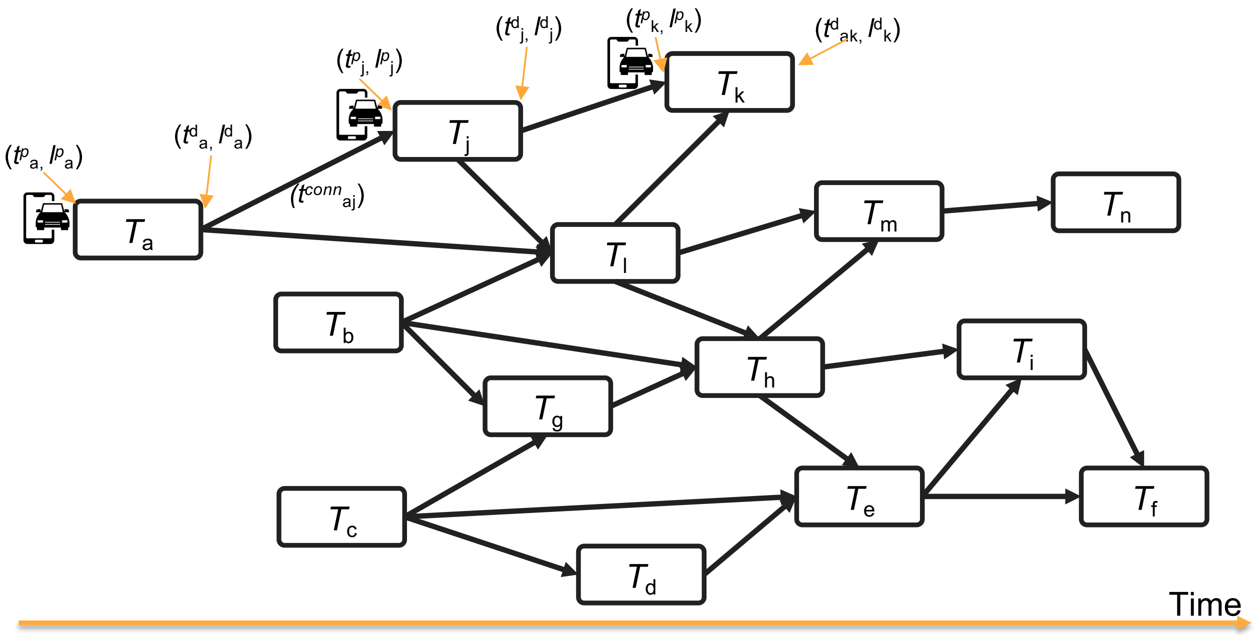

Supplementary Figure 6. Vehicle Shareability Network: a directed acyclic graph.

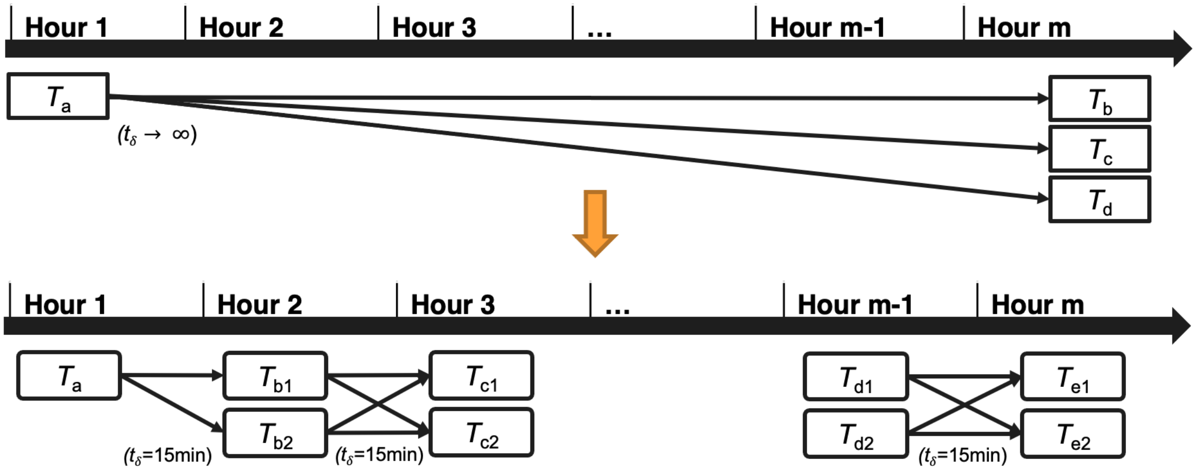

Supplementary Figure 7. Stage 1 complexity control: Limit trip connect time to 15 min so that only neighboring trips are connected, both spatially and temporally.

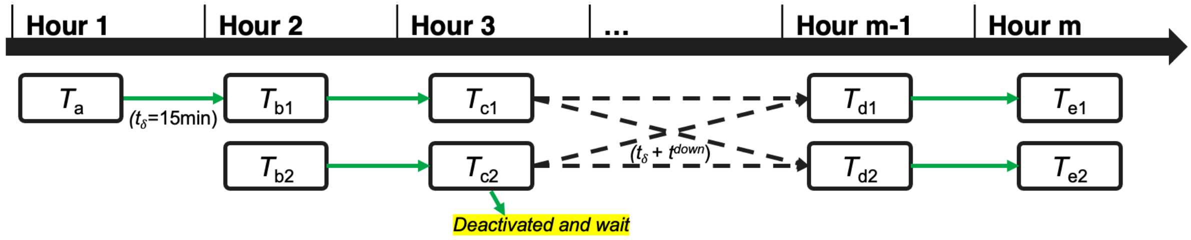

Supplementary Figure 8. Stage 2 complexity control: deactivate AEVs to sleep and allow large time window to enable trips service that are temporally distant.

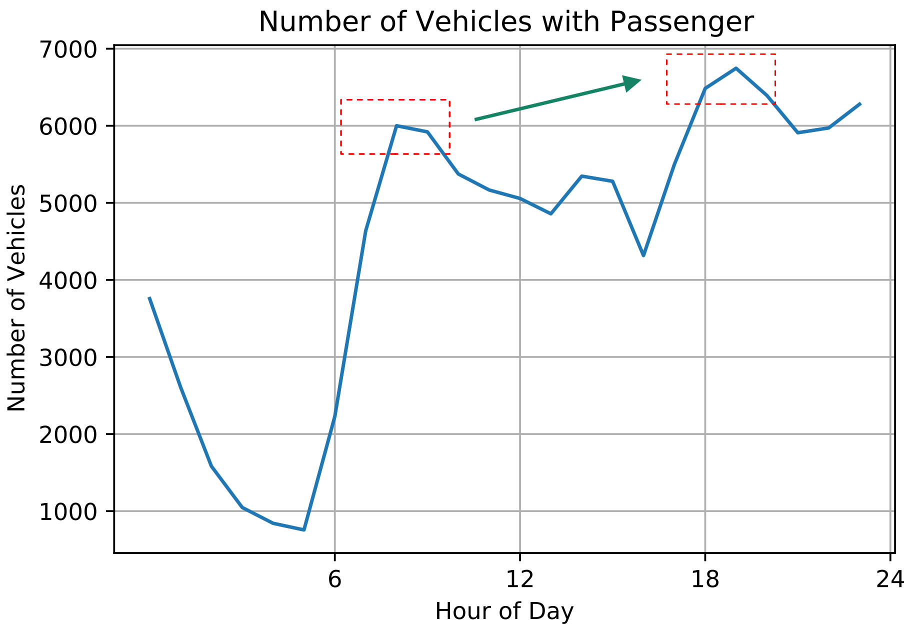

Supplementary Figure 9. Daily dual demand peaks.

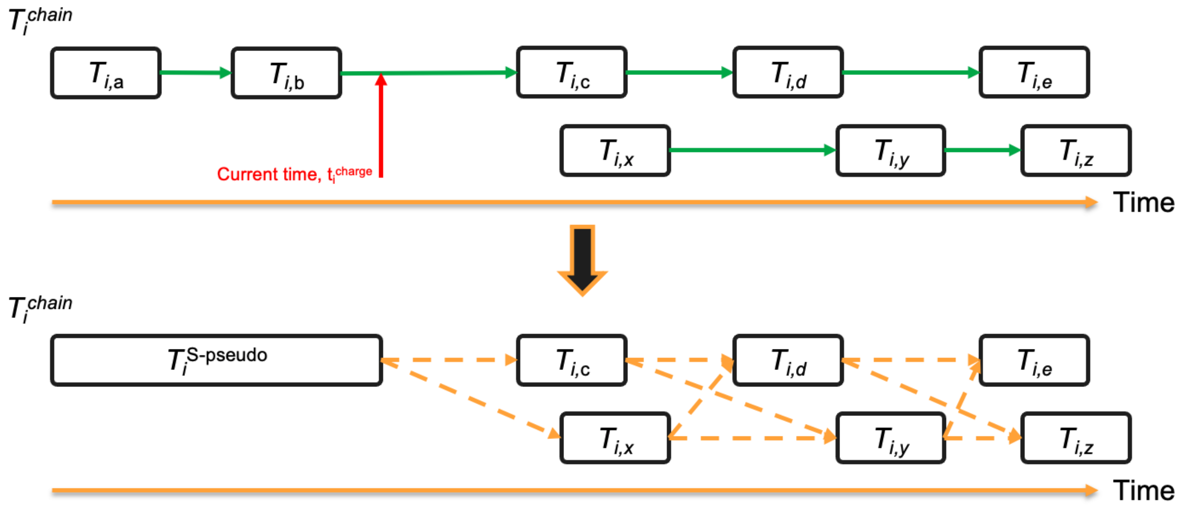

Supplementary Figure 10. Reconstruct vehicle-shareability network. Green solid lines represent the constructed trip chain in the previous iteration. Orange dashed lines represent the feasible subsequent trips and a new vehicle-shareability network was constructed. The new trip chain decisions were to be determined.

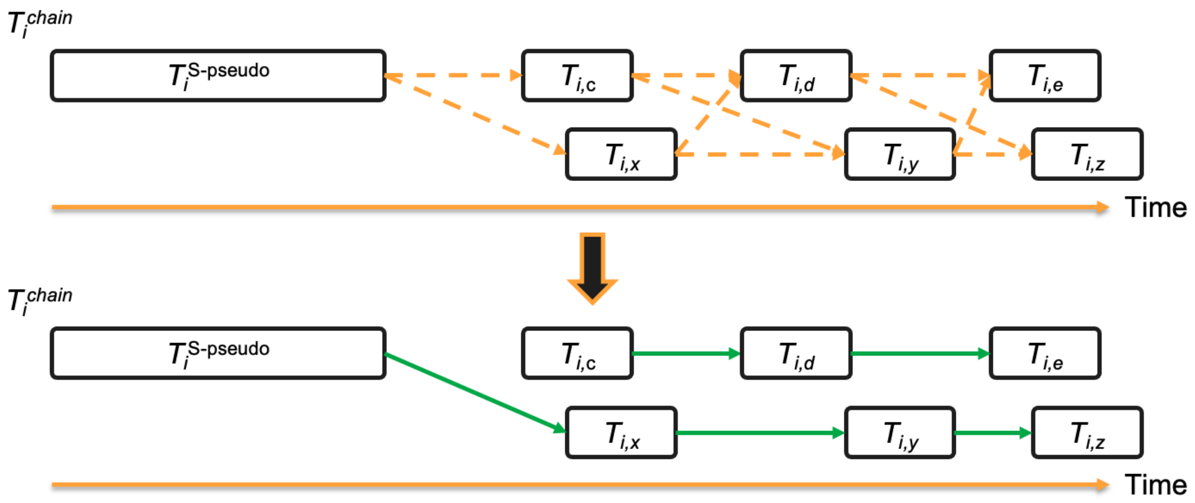

Supplementary Figure 11. Re-optimize the newly constructed vehicle-shareability network. Green solid lines represent the new determined trip chain.

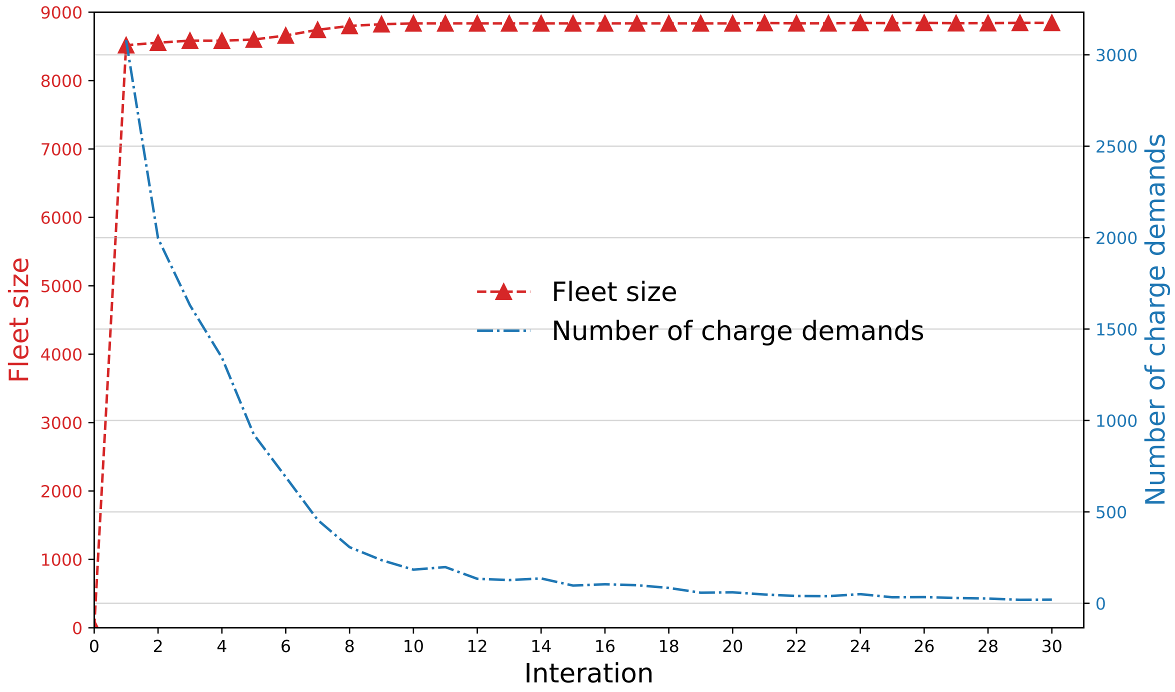

Supplementary Figure 12. Convergence analysis, it is guaranteed to converge in $O(|T||\mathscr{E}||\mathscr{N}|^{1/2})$.

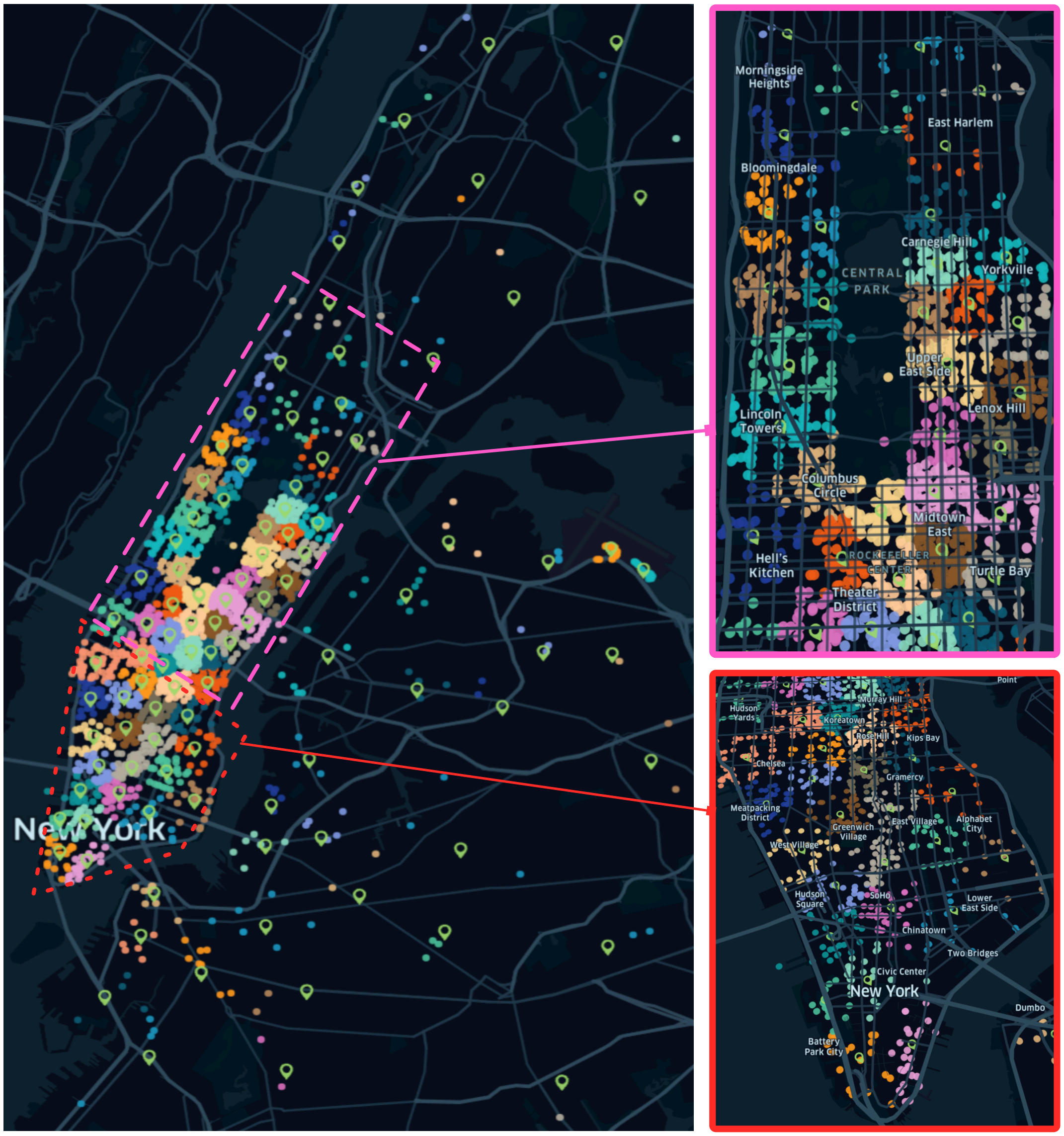

Supplementary Figure 13. Geo-spatial distribution of charging infrastructure with parameter settings: 50 kWh battery AEVs and 50 kW chargers. Each green pin icon represents a sited charging station at the centroid of a cluster. The colored / clustered dots indicate locations where AEVs were signaled to route to a charging station. The number of required chargers were placed according to the peak charging demands to ensure quality of service. Figure generated with Kepler.gl (Kepler.gl. Version (0.3.2). Retrieved from https://github.com/keplergl/kepler.gl.)

Input Names	Values	References
Annual discount factor	0.06	6
Battery cost	\$200/kWh plus 30% fleet discount	7
CO2 emission (Well-to-wheel)	2.59 gCO2/mile	8
CO2 lifecycle emission - Hydro gas	26 tonne CO2e/GWh	9
CO2 lifecycle emission - Natural gas	499 tonne CO2e/GWh	9
CO2 lifecycle emission - Nuclear	29 tonne CO2e/GWh	9
CO2 lifecycle emission - Wind	26 tonne CO2e/GWh	9
Cost of charger	\$700/charger/kW + \$15/charger/kW/year	7
Cost of station	\$10 000 per location	7
Cost of electricity	\$0.12/kWh	7
Cost of vehicle automation	\$10 000 per vehicle	7
Cost of vehicle maintenance	\$0.04/mi	7
Cost of vehicle purchase	\$20 000 per vehicle	7
Effect Factor (EF)	290 DALY/kg PM2.5 inhaled	10
Emission Factor - brake (urban)	4.7 mg PM2.5/km-vehicle	11
Emission Factor - road abrasion (urban)	4.2mg PM2.5/km-vehicle	11
Emission Factor - tyre (urban)	6.1 mg PM2.5/km-vehicle	11
Emission Factor - tailpipe (light duty vehicle)	1.86 mg PM2.5/km-vehicle	12
Fuel efficiency	0.25 kWh/mi + 0.0006 kWh/mi per kWh battery capacity	7
Intake fraction	48 ppm	13
System horizon	20 years	7
Value of Statistical Life [1] (VSL)	location = 0, scale = 7.75, shape = 1.51 (million USD)	14

Supplementary Table 1. Key parameters.

References

1. Statista Research Department. Median household income in New York from 1990 to 2019. https://www.statista.com/statistics/205974/median-household-income-in-new-york/#statisticContainer (2021). [Online; accessed 19-Sept-2022].
2. Income by Zip Code. Income statistics for New York ZIP codes. https://www.incomebyzipcode.com/newyork (2023). [Online; accessed 26-July-2023].
3. Reed, E. The average salary in New York City. https://www.yahoo.com/now/average-salary-york-city-170703352.html (2019). [Online; accessed 19-Sept-2022].
4. United States Environmental Protection Agency. Mortality risk valuation. https://www.epa.gov/environmental-economics/mortality-risk-valuation#means (2023). [Online; accessed 19-Apri-2023].
5. Colmer, J. What is the meaning of (statistical) life? benefit–cost analysis in the time of covid-19. *Oxf. Rev. Econ. Policy* **36**, S56–S63 (2020).
6. Zhang, H., Moura, S. J., Hu, Z., Qi, W. & Song, Y. A second order cone programming model for planning PEV fast-charging stations. *IEEE Trans. Power Syst.* **33**, 2763–2777, DOI: 10.1109/TPWRS.2017.2754940 (2017). 1702.01897.
7. Bauer, G. S., Greenblatt, J. B. & Gerke, B. F. Cost, energy, and environmental impact of automated electric taxi fleets in Manhattan. *Environ. Sci. Technol.* **52**, 4920–4928 (2018).
8. Sims, R. *et al.* Transport. *Clim. Chang. 2014: Mitig. Clim. Chang. Contribution Work. Group III to Fifth Assess. Rep. Intergov. Panel on Clim. Chang.* (2014).
9. Emissions, G. G. Comparison of lifecycle greenhouse gas emissions of various electricity generation sources (2011).
10. Fantke, P. *et al.* Global effect factors for exposure to fine particulate matter. *Environ. science & technology* **53**, 6855–6868 (2019).
11. Beddows, D. C. & Harrison, R. M. Pm10 and pm2. 5 emission factors for non-exhaust particles from road vehicles: Dependence upon vehicle mass and implications for battery electric vehicles. *Atmospheric Environ.* **244**, 117886 (2021).
12. United States Environmental Protection Agency. Light duty vehicle emissions. https://www.epa.gov/greenvehicles/light-duty-vehicle-emissions (2023). [Online; accessed 19-Apri-2023].
13. Apte, J. S., Bombrun, E., Marshall, J. D. & Nazaroff, W. W. Global intraurban intake fractions for primary air pollutants from vehicles and other distributed sources. *Environ. science & technology* **46**, 3415–3423 (2012).
14. Miller, T. R. Variations between countries in values of statistical life. *J. transport economics policy* 169–188 (2000).
